# Cognitive Stress Reduces the Effect of Levodopa on Parkinson's Resting Tremor

**DOI:** 10.1111/cns.12670

**Published:** 2017-01-10

**Authors:** Heidemarie Zach, Michiel F. Dirkx, Jaco W. Pasman, Bastiaan R. Bloem, Rick C. Helmich

**Affiliations:** ^1^ Department of Neurology Donders Institute for Brain, Cognition and Behaviour Radboud University Medical Centre Nijmegen The Netherlands; ^2^ Department of Neurology Medical University of Vienna Vienna Austria

**Keywords:** Cognitive co‐activation, Levodopa effect, Parkinson's disease, Tremor

## Abstract

**Aims:**

Resting tremor in Parkinson′s disease (PD) increases markedly during cognitive stress. Dopamine depletion in the basal ganglia is involved in the pathophysiology of resting tremor, but it is unclear whether this contribution is altered under cognitive stress. We test the hypothesis that cognitive stress modulates the levodopa effect on resting tremor.

**Methods:**

Tremulous PD patients (n = 69) were measured in two treatment conditions (OFF vs. ON levodopa) and in two behavioral contexts (rest vs. cognitive co‐activation). Using accelerometry, we tested the effect of both interventions on tremor intensity and tremor variability.

**Results:**

Levodopa significantly reduced tremor intensity (across behavioral contexts), while cognitive co‐activation increased it (across treatment conditions). Crucially, the levodopa effect was significantly smaller during cognitive co‐activation than during rest. Resting tremor variability increased after levodopa and decreased during cognitive co‐activation.

**Conclusion:**

Cognitive stress reduces the levodopa effect on Parkinson's tremor. This effect may be explained by a stress‐related depletion of dopamine in the basal ganglia motor circuit, by stress‐related involvement of nondopaminergic mechanisms in tremor (e.g., noradrenaline), or both. Targeting these mechanisms may open new windows for treatment. Clinical tremor assessments under evoked cognitive stress (e.g., counting tasks) may avoid overestimation of treatment effects in real life.

## Introduction

Tremor in Parkinson's disease (PD) is a highly heterogeneous and, for many patients, an agonizing symptom. It is often the first presenting sign of PD and is ranked as the second most troublesome symptom by patients in early phases of the disease 1. Many patients complain about worsened tremor under stressful circumstances in daily life 2. This may create a feeling of stigmatization and embarrassment, often leading to a vicious circle 3. Tremor also spontaneously ‘waxes and wanes’ 4, making it an unpredictable symptom both for patients and for clinicians. The neural mechanisms that contribute to tremor amplification during stress, and also to the spontaneous variations in tremor, are unclear. Having such insights would be helpful for the development of improved treatment strategies for tremor. In this study, we address this issue by testing whether the effect of levodopa on resting tremor is modulated by the presence of acute cognitive stress.

The pathological hallmark of PD is nigrostriatal dopamine depletion 5, but the dopaminergic basis of resting tremor is disputed 6. Specifically, striatal dopamine depletion correlates with all motor symptoms except resting tremor 7, and dopaminergic medication has a variable (and sometimes no) effect on resting tremor 8, 9. This suggests that other neurotransmitter systems may play an (additional) role in the pathophysiology of PD resting tremor. Accordingly, nuclear imaging studies have shown a correlation between tremor severity and serotonin depletion in the raphe 10, 11. Furthermore, there is evidence for a role of the noradrenergic system in PD resting tremor: Tremor‐dominant PD patients have *less* degeneration of the locus coeruleus (the main source of cerebral noradrenalin) than nontremor patients 12, and intravenous injection of adrenalin increases tremor 13. Indeed, it has been argued that the increase in tremor during cognitive stress could be related to activation of the locus coeruleus–noradrenergic system 14. Taken together, these findings suggest that different neurotransmitter systems may play a variable, context‐specific role in the pathophysiology of PD resting tremor.

Here, we test the hypothesis that the contribution of dopamine depletion to PD resting tremor is modulated by acute cognitive stress (when the noradrenergic system is activated). Using accelerometry, we measured tremor intensity (maximal tremor power during each condition) and tremor variability (coefficient of variation of tremor power) at rest and during cognitive stress (serial sevens), both OFF and ON dopaminergic medication. We used a mental arithmetic task under social evaluation (by the examiner), because previous studies have shown that this task induces autonomic stress responses 15 and is associated with activity in a cerebral stress circuit 16. We expected a diminished levodopa effect during cognitive stress. Furthermore, we hypothesized to find higher, but more stable, tremor intensity during cognitive stress.

## Materials and Methods

### Study Population

Patients with PD, diagnosed according to the UK Brain bank criteria 17, 18, were included. Patients without current dopaminergic medications had to meet at least 3 of the “supportive UK Brain bank criteria” (unilateral onset, rest tremor, and progressive disorder). We only included patients with a resting tremor score of ≥1 points (pts.) in at least one arm on item 17 of the Movement Disorders Society‐Unified Parkinson's Disease Rating Scale (MDS‐UPDRS). All patients were recruited from our outpatient clinic and examined by two independent movement disorders specialists. Exclusion criteria were as follows: neurological comorbidity, signs of psychogenic tremor (e.g., entrainment or distractibility), known allergy against levodopa–benserazide or domperidone, and significant cognitive impairment (Mini‐Mental State Examination (MMSE) score <24 or frontal assessment battery (FAB) < 12) 19, 20.

The study was approved by the Local Ethics Committee and was performed according to the standards of the 1964 Declaration of Helsinki. All participants gave written informed consent prior to their inclusion. Patients were clinically assessed using the MDS‐UPDRS‐part III 21 and the Hoehn and Yahr Scale (H&Y) 22.

### Design

Patients were measured twice on 1 day, both before (OFF medication) and after a levodopa challenge (ON medication). We quantified clinical characteristics using the MDS‐UPDRS and electrophysiological tremor characteristics (maximal tremor intensity and tremor variability) using accelerometry. Cognitive characteristics were measured once, using the MMSE and the FAB.

### Levodopa Challenge

All patients were evaluated after overnight fasting and in a practically defined OFF state, more than 12 h after intake of their last dose of levodopa, more than 30 h after dopaminergic agonists, and more than 24 h after anticholinergics or beta‐blockers 23. To avoid possible influence on tremor patients had to forgo caffeine (tea, coffee) for >12 h. For the ON state assessment, the patients first received 10 mg domperidone to reduce possible side effects and to improve gastrointestinal absorption. This was followed by a standard dose of 250 mg dispersible levodopa–benserazide (on average 75% higher than the patients' own morning dose) 1 h later 23.

### Tremor Assessment

Clinical resting tremor was assessed with the MDS‐UPDRS motor scale (part III) using items 17 (tremor amplitude of the most affected hand) and item 18 (tremor constancy, i.e., percentage of visible rest tremor during the entire examination; range 0 to >75%) 21. The clinical rater was present during the entire examination and rated tremor clinically during the entire session, including the electrophysiology part, breaks between the tasks, and the whole MDS‐UPDRS part III examination in OFF and in ON state. Two different tremor features (of the most affected arm) were quantified using accelerometry: tremor intensity (maximal tremor power during each condition) and tremor variability (coefficient of variation of tremor power; see section Statistical Analysis). For better comparability with clinical ratings, the maximal (rather than mean) tremor intensity was quantified with the log values of the amplitudes, derived by accelerometry, because MDS‐UPDRS item 17 is based on the highest amplitude at any time during the entire examination. Patients lied down comfortably on a bed to achieve complete resting state. The forearms rested in a relaxed position on two pillows, while hands and fingers were unsupported. Resting tremor was recorded in two different contexts: at rest (REST) and during cognitive co‐activation (COCO); backwards counting in steps of three or seven as fast as possible, while the examiner verbally encouraged rapid responding (social evaluation) 24. For each context, we collected three trials of 60 s each.

### Accelerometer Data Analysis

ECG was measured to calculate heart rate during each condition. Resting tremor intensity was measured with a lightweight biaxial piezoelectric accelerometer (Medifactory international; Heerlen, the Netherlands; sensitivity: 128 Hz) attached to the dorsum of the most affected hand. Data were stored on a computer for offline analysis. For preprocessing, a bandpass filter of 1–40 Hz was used to remove slow‐frequency drifts and high frequencies of no interest. Next, we analyzed the data using FieldTrip 25. Specifically, we calculated the time–frequency representations (TFR) between 1 and 20 Hz using a 2 s. Hanning taper, which resulted in a 0.5 Hz resolution. By averaging over all time points, we obtained an average power spectrum across segments. For each patient, we picked the TFR of the corresponding tremor frequency, resulting in patient‐specific regressors describing fluctuations in tremor intensity during the 60‐s trial. After log‐transformation, we calculated maximal tremor intensity by taking the highest value of the tremor regressor (for each patient, context, and trial). This value reflects the highest tremor episode (based on a 2‐s sliding window) during each trial. Furthermore, we calculated variability of tremor intensity using the coefficient of variation (mean of the tremor regressor divided by its standard deviation).

### Statistical Analysis

First, we compared the clinical characteristics (including heart rate) OFF versus ON levodopa using paired *t*‐tests (two‐sided). Second, we compared the electrophysiological resting tremor characteristics (1) maximal resting tremor intensity, and (2) tremor variability using two‐way repeated‐measures analysis of variance (ANOVA) with factors TREATMENT (OFF vs. ON levodopa) and CONTEXT (REST vs. COCO). Third, we correlated clinical characteristics and electrophysiological tremor characteristics, and we correlated the dopamine response (OFF vs. ON, averaged over REST and COCO) with the context response (COCO vs. REST, averaged over OFF and ON), using a Spearman correlation (two‐sided). All statistical analyses were performed using SPSS (IBM SPSS Statistics for Windows, Version 22.0, IBM Corp., Armonk, NY, USA).

## Results

A total of 71 patients (49 men; mean age: 63 years; range: 45–81) were recruited from our outpatient clinic and examined by two independent movement disorders specialists. The recruited patients were moderately affected (indicated by a median HY stage of 2), and the overall results of cognitive tests (MMSE and FAB) indicated good cognitive functioning. Of 71 patients, two were excluded (signs of psychogenic and atypical tremor). All analyses were performed on the remaining 69 patients. Eight patients did not use anti‐Parkinson medication, and the others used dopaminergic medication at home (levodopa and dopamine agonists; average daily levodopa equivalent: 439.5 mg; range: 0–1500 mg). Six patients were on anticholinergics. Eight patients used beta‐blockers (for hypertension and/or tremor). Patient characteristics are listed in Table 1.

**Table 1:** Clinical characteristics

Age (years; mean)	63 (45–81)
Sex	20 F, 49 M
HY stage (median)	2.0 (1.0–3.0)
Disease duration (years; mean)	3.9 (0.3–15)
MMSE (mean)	29 (24–30)
FAB (mean)	17 (13–18)
Levodopa equivalent at home (mg/day; mean)	440 (0–1500)

H&Y stage, Hoehn and Yahr stage (score 0–5); MMSE, Mini‐Mental State examination (score 0–30); FAB, frontal assessment battery (score 0–18).

If not indicated otherwise, data are mean (±standard error of the mean) across 69 Parkinson patients.

For HY stage, higher scores indicate worse functioning. For both FAB and MMSE, lower scores indicate worse functioning. The scores were evaluated OFF medication.

The first tremor assessment in ON state started on average 47 min (range: 35–59 min) after taking levodopa. Levodopa had a significant effect on clinical measures of disease severity, including resting tremor (Table 2). The average tremor frequency was 4.78 (±0.08) Hz and was not affected by either TREATMENT or CONTEXT (*P* > 0.19). Heart rate significantly *increased* during COCO (average at REST: 63 ± 1.2 bpm, during COCO: 71 ± 1.5 bpm; main effect of CONTEXT, F(1,68) = 31.2; *P* < 0.001), but there was no significant effect of treatment (TREATMENT, F(1,68) = 3.2, *P* = 0.08), and there were no significant interactions (F(1,68) = 0.4, *P* = 0.53). This suggests that patients experienced cognitive stress during COCO.

**Table 2:** Clinical Levodopa effect (MDS‐UPDRS part III)

	OFF state	ON state	Improvement	Significance (*P*)
UPDRS part III OFF state (mean)	43.9 (±1.80)	25.9 (±1.38)	41.3% (±1.79)	<0.001
Tremor score MA hand (item 17; mean)	2.97 (1–4)	2.01 (0–4)	35.0% (±4.12)	<0.001
Tremor constancy (item 18; mean)	3.26 (1–4)	1.70 (0–4)	47.3% (±4.20)	<0.001

MDS‐UPDRS, Movement Disorder Society‐Unified Parkinson's disease rating scale part III (score 0–132; item 17 ranges from 0 to 20); OFF state, without dopaminergic medication; ON state, after dopaminergic medication; MA hand, most affected hand.

Data are mean (±standard error of the mean) across 69 Parkinson patients. Higher scores indicate worse symptoms.

### Levodopa Effect on Tremor Intensity

**Figure 1:**
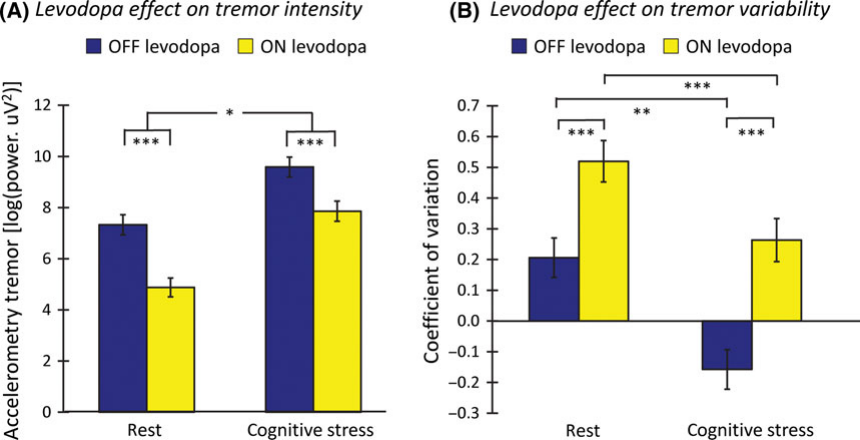
Levodopa effect on resting tremor. Panel **A** shows the levodopa effect on tremor intensity (maximal tremor power during each condition, measured with accelerometry) during REST (left) and COCO (right) in OFF and ON levodopa state (colored bars). Levodopa significantly reduced resting tremor intensity, while COCO significantly increased it. However, the effect of levodopa was significantly smaller during COCO than REST. Panel **B** shows the levodopa effect on tremor variability (coefficient of variation of tremor power) during REST and COCO. Levodopa significantly increased tremor variability, while cognitive co‐activations reduced it. Level of significance: *P < 0.05; **P < 0.01; ***P < 0.001; data are log‐transformed. COCO, cognitive co‐activation.

Levodopa and cognitive co‐activation had opposite effects on resting tremor intensity: Levodopa significantly *reduced* maximal tremor intensity (main effect of TREATMENT, F(1,68) = 42.9; *P* < 0.001), while cognitive co‐activation significantly *increased* tremor intensity (main effect of CONTEXT, F(1,68) = 128.9; *P* < 0.001). Crucially, the effect of levodopa was significantly smaller during COCO than during REST (TREATMENT × CONTEXT interaction, F(1,68) = 5.6; *P* = 0.02; Figure 1A). In other words, the positive effect of levodopa was counteracted by a negative effect of cognitive stress such that maximal tremor intensity during COCO in the ON state was similar to maximal tremor intensity during REST in the OFF state (t(68) = 1.4; *P* = 0.2). *Post hoc t*‐tests revealed that the effect of levodopa was present for both REST (t(68) = 6.9; *P* < 0.001) and COCO (t(68) = 4.9; *P* < 0.001) conditions and that cognitive co‐activation increased tremor intensity for both OFF (t(68) = 8.7; *P* < 0.001) and ON (t(68) = 10.2; *P* < 0.001) sessions. The effects of levodopa (OFF > ON) and context (COCO > REST) appeared to be independent from each other: There was no correlation between both difference scores (Spearman's rho = 0.12, *P* = 0.31).

### Levodopa Effect on Tremor Variability

Levodopa significantly *increased* tremor variability (main effect of TREATMENT, F(1,68) = 43.6, *P* < 0.001; Figure 1B), while cognitive co‐activation *decreased* tremor variability (main effect of CONTEXT, F(1,68) = 32.2, *P* < 0.001). There was no significant interaction between the conditions (F(1,68) = 1.4, *P* = 0.24). This finding indicates that patients switched more often between low‐ and high‐amplitude tremor in the ON state and at rest, while they experienced a more consistent (more severe) tremor in the OFF state and during cognitive stress.

### Correlation of Clinical and Accelerometry Data

**Figure 2:**
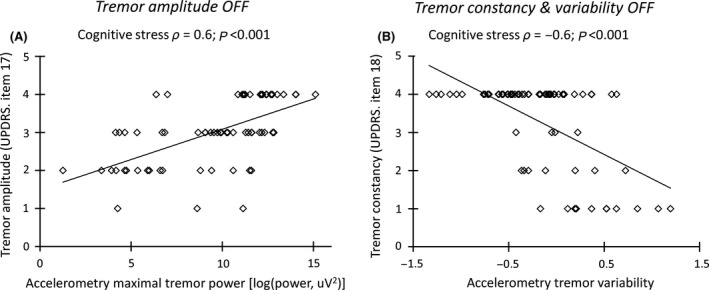
Correlation of clinical versus electrophysiological evaluation. Panel **A** shows that increased tremor intensity (maximal tremor power, measured using accelerometry; *x*‐axis) was associated with increased clinical tremor severity (MDS‐UPDRS item 17 for the most affected hand, range from 0 to 4 points; *y*‐axis), during cognitive co‐activation in the OFF state. Panel **B** shows that increased tremor variability (coefficient of variation of tremor power, measured using accelerometry; *x*‐axis) was associated with reduced clinical tremor constancy (MDS‐UPDRS item 18; range from 0 to 4 points; *y*‐axis) during cognitive co‐activation in the OFF state.

As expected, electrophysiological and clinical tremor characteristic were strongly related. First, maximal resting tremor intensity showed a significant and positive correlation with clinical tremor severity (MDS‐UPDRS, item 17), both OFF levodopa (REST: *ρ* = 0.5; *P* < 0.001; COCO: *ρ* = 0.6; *P* < 0.001; see Figure 2A) and ON levodopa (REST: *ρ* = 0.4; *P* < 0.001; COCO: *ρ* = 0.5; *P* < 0.001). Second, maximal resting tremor intensity and the clinical tremor constancy (item 18) were significantly correlated, both OFF levodopa (REST: *ρ* = 0.6; *P* < 0.001; COCO: *ρ* = 0.5; *P* < 0.001) and ON levodopa (REST: *ρ* = 0.5; *P* < 0.001; COCO: *ρ* = 0.4; *P* < 0.01). Third, the reduction in tremor intensity, which was clinically (item 17) and electrophysiologically detected, significantly correlated during COCO (*ρ* = 0.4; *P* < 0.001). Finally, we observed a negative correlation between resting tremor variability (using accelerometry) and tremor constancy (item 18), both in the OFF state (REST: *ρ* = −0.5; *P* < 0.001; COCO: *ρ* = −0.6; *P* < 0.001; see Figure 2B) and in the ON state during COCO (*ρ* = −0.6; *P* < 0.001).

## Discussion

We tested the hypothesis that the antitremor effect of levodopa is reduced during cognitive stress. The cognitive co‐activation task activated the noradrenergic stress system, as evidenced by a significant increase in heart rate 15. Our main finding is that cognitive stress reduced the ability of levodopa to suppress tremor intensity, as compared to a resting condition. Furthermore, we found that levodopa increased the spontaneous variability of resting tremor amplitude, while cognitive stress decreased it.

Our findings appear to contradict a previous study by Sturman and colleagues, who reported similar effects of levodopa on tremor intensity for tremor at rest and for tremor during cognitive co‐activation in 10 PD patients 26. However, this discrepancy might be explained by methodological differences: Sturman et al. only included patients with implanted deep brain stimulation electrodes in the STN, and both the patient characteristics (surgical candidates differ from the general PD population) and micro‐lesions induced by the surgery may have altered the levodopa response. Furthermore, while their cognitive co‐activation task was externally paced, we aimed to induce higher stress level by instructing to “count as fast as possible” (i.e., internally paced) 27. Finally, our sample was considerably larger than the previous study (69 vs. 10 patients).

### Possible Mechanisms Underlying the Reduced Levodopa Effect on Tremor During Cognitive Stress

The finding that levodopa had a relatively reduced antitremor effect during cognitive stress may be explained by the involvement of nondopaminergic mechanisms, or by a depletion of available dopamine during cognitive stress, or both. Our task reliably activated the noradrenergic stress system, as evidenced by a significant increase in heart rate 15, 27. Although it is known that the noradrenergic system is affected in early stage PD 28, neuropathological studies revealed a high association of locus coeruleus degeneration with akinetic‐rigid phenotypes, but only minimal involvement of the locus coeruleus in tremor‐dominant patients 12, 14. This suggests that an intact noradrenergic system may be necessary for the development of tremor, although there is no evidence for a causal link. Animal studies have shown that an acute stressor produces motor hyperactivity, possibly mediated through direct effects of noradrenalin onto the cerebral motor circuit 29. Using fMRI, we have previously shown that PD resting tremor is triggered by the dopamine‐depleted basal ganglia, and amplified in the cerebello‐thalamo‐cortical circuit 30. The locus coeruleus sends noradrenergic projections to all nodes of the cerebello‐thalamo‐cortical circuit 14, 27, which may amplify tremulous activity in this circuit.

Another possibility is that cognitive stress depleted available dopamine in tremor‐related regions of the basal ganglia and that our dopaminergic intervention was insufficient to replete this functional deficit. More specifically, it is conceivable that our (cognitive) task leads to a redistribution of dopamine from motor to cognitive portions of the cortico‐striatal circuit, causing dopamine depletion in the cortico‐striatal motor loop. On the other hand, our dopaminergic intervention (levodopa/benserazide 200/50 mg) was considerably larger than the dose taken by most patients. This makes underdosing unlikely—even in the presence of cognitive co‐activation. Neuroimaging studies may further test whether cognitive stress increases PD tremor by activating the noradrenergic system, by depleting the dopaminergic system, or both.

Finally, it is also unlikely that our task increased tremor through the peripheral nervous system, given the immediate effect of cognitive stress on tremor intensity (i.e., in less than a second). Furthermore, previous work has shown that adrenalin administration only increases Parkinson's tremor when injected intravenously (i.e., systemically, enabling central effects 31), but not when injected into an artery—which distributes adrenalin to the muscle 32. It is also unlikely that psychological stress modulates tremor through spinal mechanisms, given absent effects of cognitive stress on the Hoffmann's reflex 33, 34.

### Tremor Variability

Tremor variability was highest during the ON‐REST state and lowest during the OFF‐COCO state. A possible explanation for the high variability ON levodopa is that levodopa desynchronizes oscillatory activity in the basal ganglia, leading to more irregular tremor frequencies 26. Since frequency changes are associated with (temporary) amplitude reductions, this may have lead to higher amplitude variability in ON state 35. High tremor variability may continuously bring this symptom under the patient's attention, thereby contributing to a subjective experience of many patients that levodopa is ineffective in reducing resting tremor, whereas in fact the tremor amplitude is lower.

### Clinical Implications

The decreased levodopa effect during cognitive stress underlines the importance of using simple (rapid) counting tasks for clinically assessing tremor 24. Although there are several tremor provocation tasks, the serial‐sevens task used here is one of the most practicable ones 24. Otherwise, it is possible that the clinical examination underestimates tremor intensity and, importantly, potentially overestimates the effect of levodopa and therefore does not reflect the patient's tremor burden in daily life. Ideally, tremor‐rating scales should describe explicitly under which circumstances the tremor was scored, because the context obviously has a major effect on tremor severity. Currently, the resting tremor rating in MDS‐UPDRS part III (item 17) only scores the maximal tremor amplitude in the context chosen by the investigator.

Our findings highlight an important clinical problem: Patients suffer most from tremor in stressful circumstances, when available (dopaminergic) therapy is least effective. This calls for new treatments aimed at reducing the influence of acute stress on resting tremor. This may be achieved by cognitive behavioral therapy 36 or pharmacological therapy that blocks noradrenergic transmission (e.g., beta‐blockers).

Our findings may also have implications for future studies aimed at *enhancing* noradrenergic activity in PD. Specifically, it has been suggested that noradrenergic enhancement may alleviate symptoms such as apathy, cognitive dysfunction, freezing of gait, and sleep disorders 37. Our findings raise the possibility that noradrenergic enhancement is not suitable for a subset of patients that are particularly sensitive to the effects of acute cognitive stress on resting tremor.

### Interpretational Issues

We did not collect a direct measure of activity in the locus coeruleus–noradrenergic system. Thus, although our task was associated with increased heart rate, which is associated with noradrenergic activity 15, we cannot rule out an additional effect of distraction, concentration, or speaking—as in previous studies 38. Also, our findings do not suggest that noradrenergic activity is the *only* factor that modulates tremor intensity: Motor co‐activation (such as walking or finger tapping with the hand contralateral to the tremor) can also increase tremor, possibly through other mechanisms 24.

We only measured electrophysiological tremor severity (with accelerometry) on the most affected arm. Therefore, it remains unclear whether our findings can be extrapolated to tremor in other body parts—although it is unlikely that tremors in different body parts have a different pathophysiology.

All patients were aware of the intervention, and therefore, a placebo effect may have contributed to the tremor reduction in the ON state. However, an influence on our main finding (interaction effect with context) is unlikely, since a placebo effect would have been similar for both contexts. For the same reason, it is unlikely that our findings are influenced by order effects (ON was always after OFF).

Finally, given the absence of validated context‐specific tremor‐rating scales, we do not have clinical ratings separately for the REST and COCO conditions 39. This makes it difficult to judge the clinical relevance of our accelerometry findings.

## Conclusion

Our results show a reduced levodopa effect in PD resting tremor during cognitive stress and lower tremor variability in this context. Both findings have clinical relevance: They emphasize the impact of simple counting tasks in clinical practice to avoid overestimation of treatment effects visible at rest. Mechanistically, our findings suggest that not only dopaminergic, but also nondopaminergic, circuits may be involved in the pathophysiology of Parkinson's tremor—and that the contribution of different circuits may be context‐specific. Finally, our findings open new windows for personalized treatment of Parkinson's tremor, taking patient‐ and context‐specific factors into account.

## Conflict of Interest

The authors declare no conflict of interest.
